# Establishment and Characterization of an Aβ-Related Alzheimer’s Disease-like Tree Shrew Model Following CA1-Coordinate–Directed Stereotaxic AAV Delivery of Human Triple-Mutant APP

**DOI:** 10.3390/biology15131071

**Published:** 2026-07-04

**Authors:** Yixuan Yang, Qiurui Li, Shaoshi Luo, Junming Sun, Yiqiang Ouyang

**Affiliations:** 1 Laboratory Animal Center, Guangxi Medical University, Nanning 530021, China; 202320004@sr.gxmu.edu.cn (Y.Y.); qiurui6089@163.com (Q.L.); huanying0771@163.com (S.L.); 2 School of Basic Medical Sciences, Guangxi Medical University, Nanning 530021, China; 3 China–ASEAN Laboratory Animal Science and Technology Innovation Center, Guangxi Medical University, Nanning 530021, China

**Keywords:** Alzheimer’s disease, tree shrew, amyloid-β, APP, animal model

## Abstract

Alzheimer’s disease (AD) is characterized by cognitive decline and amyloid-β (Aβ)-related pathology. Non-rodent models that capture selected aspects of human AD remain limited. We established and characterized a human APP-driven, Aβ-related AD-like tree shrew model following AAV-mediated delivery of triple-mutant human amyloid precursor protein (hAPP-SLA) carrying the Swedish, Austrian, and London mutations by bilateral stereotaxic injection directed at CA1 coordinates. Adult tree shrews received bilateral AAV-hAPP-SLA injections directed at CA1 coordinates and were evaluated by bioluminescence imaging, behavioral testing, PCR, RT-qPCR, Western blotting, ELISA, and histopathology. Vector-associated reporter signals remained detectable for 6 months. The experimental group showed exogenous hAPP expression and reduced endogenous tsAPP expression, increased relative hippocampal Aβ_42_ protein level, enhanced 4G8-reactive APP/Aβ-related signals, elevated total Aβ immunoreactivity, increased serum Aβ_42_/Aβ_40_ ratio, cytoarchitectural alterations, reduced Nissl staining, and Thioflavin S-reactive aggregate-associated signals. AT8 (Ser202/Thr205), GFAP, and Iba-1 immunoreactivity increased, whereas Synaptophysin and PSD-95 immunoreactivity was reduced. These changes were accompanied by reduced short-delay recognition-related performance and reduced social approach and social novelty preference. Aged tree shrews showed partly overlapping alterations. This model provides a non-rodent platform for studying human APP-driven Aβ-related pathology.

## Simple Summary

Alzheimer’s disease causes memory problems, changes in thinking, and behavioral symptoms. Rodents are widely used in research, but they do not reproduce all features of human disease. Non-human primates are more similar to humans but require substantial time, resources, and ethical oversight. Tree shrews are small non-rodent mammals with several brain and behavioral characteristics relevant to primate biology and may provide a complementary model between rodents and primates. In this study, we delivered a human APP gene carrying three Alzheimer’s disease-linked mutations to adult tree shrews by bilateral stereotaxic injection directed at CA1 coordinates using a viral vector. We assessed gene expression, amyloid-beta-related molecular changes, brain pathology, and behavior. The experimental animals showed expression of the introduced human APP gene, amyloid-beta-related and amyloid-like pathological changes, Tau-related changes, glial-cell responses, altered synaptic-marker staining, and changes in short-delay recognition-related and social behavior. This model may be useful for investigating human APP-driven amyloid-beta-related processes in Alzheimer’s disease research.

## 1. Introduction

Alzheimer’s disease (AD) is among the most common neurodegenerative disorders and is clinically associated with progressive memory impairment, cognitive decline, and changes in behavior and social interaction. As population aging accelerates worldwide, AD has become a major cause of disability in older adults and has placed an increasing burden on families and healthcare systems [1,2]. Pathologically, AD is defined by a series of interconnected changes, including amyloid-β (Aβ) deposition, Tau hyperphosphorylation, synaptic dysfunction, neuronal loss, and neuroinflammation [3,4,5,6,7]. Among these events, an imbalance between Aβ production and clearance is generally regarded as an early event in disease development. A higher Aβ_42_/Aβ_40_ ratio is particularly important because Aβ_42_ aggregates more readily and contributes to amyloid plaque formation. For this reason, animal models that reproduce Aβ-related pathology alongside cognitive or behavioral impairment remain essential for studying disease mechanisms and evaluating therapeutic strategies [8,9,10,11,12].

Current AD models include transgenic, toxin-induced, natural aging, and viral vector-mediated gene delivery models. Transgenic mice have been used most widely because they are genetically tractable, inexpensive to maintain, and suitable for relatively short experimental cycles. However, rodents differ from humans in brain organization, sensory dominance, behavioral complexity, and the course of neurodegenerative changes. These differences restrict the extent to which findings from rodent models can be translated to human AD [7,8,9,10]. Natural aging models provide another way to study age-related neurodegeneration, but they usually require long observation periods and may show variable or unstable Aβ pathology. Viral vector-mediated gene delivery offers a more flexible alternative by enabling selected genes to be introduced into specific brain regions and expressed within a defined experimental window. This strategy is therefore useful for inducing localized AD-like pathology within a shorter period of time. Among AAV serotypes, AAV9 has been widely used for CNS gene delivery and can support efficient neuronal transduction and durable transgene expression after direct intracranial or stereotaxic administration under appropriate promoter and delivery conditions [13,14,15]. AAV-mediated gene transfer has also been used to generate AD-related proteinopathy models in complementary experimental systems [16].

Pathogenic mutations in the amyloid precursor protein (APP) gene are closely linked to familial AD. The Swedish, London, and Austrian mutations affect APP processing and are associated with increased Aβ production, a higher Aβ_42_/Aβ_40_ ratio, and greater aggregation tendency [3,4]. Delivering human APP with multiple AD-related mutations into the brain can therefore induce APP/Aβ-associated molecular and pathological changes. Most studies using this strategy have been performed in rodents. In contrast, similar work in small mammals with greater translational relevance remains limited. There remains a need for intermediate animal models that are experimentally feasible and biologically closer to primates than conventional rodents [7,8].

The tree shrew (*Tupaia belangeri*) is a small diurnal mammal that can be maintained and tested more easily than non-human primates. Compared with rodents, tree shrews exhibit several neurobiological features more similar to those of primates [17,18,19], including aspects of brain organization, a visually dominant sensory system, and a diurnal activity rhythm [19,20,21]. The Aβ amino acid sequence of tree shrews also shows high homology with that of humans [22]. Previous studies have reported AD-like changes in aged tree shrews, including Aβ deposition, Tau hyperphosphorylation, oxidative stress, and age-related cognitive decline [23,24]. These findings support the use of tree shrews as an intermediate model between rodents and non-human primates. Previous studies have established AD-like models in tree shrews through hippocampal administration of Aβ_1–40_, reporting cognitive impairment, neuronal apoptosis, altered gene expression, and pharmacological responsiveness to donepezil [25,26]. More recently, hippocampal AAV9-mediated delivery of APPswe/MAPTp301s in tree shrews was shown to induce AD-related pathological alterations, including Aβ plaque deposition, neuronal loss, glial activation, neurofibrillary tangle-like pathology, and increased phosphorylated Tau expression [27]. These findings support the feasibility of using tree shrews for AD-related modeling. Nevertheless, APP-driven AD-like models in tree shrews remain underdeveloped, particularly those based on AAV-mediated delivery of human pathogenic APP following bilateral stereotaxic injection directed at CA1 coordinates, with integrated molecular, pathological, and behavioral assessment. Therefore, the present study aimed to establish and characterize an AAV-mediated human mutant APP tree shrew model following bilateral stereotaxic injection directed at CA1 coordinates. The hippocampus is a key brain region for learning and memory and is affected early in AD [28]. Aβ accumulation and neuronal injury in this region can impair hippocampus-dependent spatial and recognition memory. AAV-mediated delivery of human triple-mutant APP using CA1-coordinate–directed stereotaxic injection may provide a practical approach to induce Aβ-related alterations and assess their behavioral consequences. In the present study, a recombinant AAV vector carrying human APP with the Swedish, London, and Austrian mutations was stereotactically injected into the hippocampal CA1 region of tree shrews. Normal control and vector control groups were included, and an aged tree shrew group was added as a reference for age-related changes. We assessed exogenous hAPP expression, endogenous tsAPP expression, Aβ-related protein changes, the Aβ_42_/Aβ_40_ ratio, hippocampal pathology, and behavioral performance using PCR, Sanger sequencing, RT-qPCR, Western blotting, ELISA, histopathological staining, and behavioral assays. This design enabled comparison of AAV-hAPP-SLA-induced alterations with both standard controls and the older-aged animals. Accordingly, this study aimed to establish and characterize an AAV-mediated human triple-mutant APP tree shrew model with AD-like features following CA1-coordinate–directed stereotaxic injection and to examine the relationship between human APP-driven Aβ-related alterations and age-associated AD-like changes. This work may provide an experimental platform for AD model development, mechanistic studies, and therapeutic evaluation.

## 2. Materials and Methods

### 2.1. Animals and Experimental Design

A total of 24 tree shrews were included in this study, comprising 18 adult animals aged 8 months (130 ± 30 g) and 6 aged animals aged approximately 72 months at study enrollment (120 ± 20 g). The aged animals underwent the same 6-month observation period as the adult experimental cohorts and were therefore approximately 78 months old at terminal tissue collection. All animals were obtained from the Kunming Institute of Zoology, Chinese Academy of Sciences, and were kept in a standard animal facility at Guangxi Medical University. The housing conditions were arranged according to the natural diurnal rhythm of tree shrews. Animals had free access to food and water, with fresh fruit and protein supplements provided as part of the routine diet. All procedures were carried out in accordance with the 3R principles for animal welfare.

Before modeling, all tree shrews underwent behavioral prescreening based on locomotor activity, exploratory behavior, stress reactivity, and general responsiveness, with reference to previously reported baseline behavioral characteristics and neurobehavioral assessment methods in tree shrews [29]. Animals showing marked abnormal responses, including excessive stress reactions, failure to explore the testing environment, persistent inactivity, or obvious abnormalities in basic locomotor behaviors, were excluded before group allocation. Both male and female animals were included in the study. Each group, including the NC, VC, EXP, and AGED groups, comprised three males and three females (*n* = 6 per group). Eligible adult tree shrews were randomly divided into the NC, VC, and EXP groups. Data from male and female animals were pooled for group-based analysis because the sample size within each sex subgroup was limited (*n* = 3 per sex per group), and sex was not included as an independent statistical factor.

### 2.2. Reagents and Instruments

Recombinant adeno-associated viruses (rAAV-hAPP-SLA-NLuc and rAAV-NLuc) were purchased from Wuhan Shumi Brain Science Technology Co., Ltd. (Wuhan, China). The stereotaxic apparatus, anesthesia system, and behavioral tracking system (SMART 3.0) were obtained from RWD Life Science Co., Ltd. (Shenzhen, China). The in vivo imaging system was purchased from PerkinElmer (Waltham, MA, USA). The luciferase substrate Furimazine (#162434) was obtained from Nantong Quanyi Biotechnology Co., Ltd. (Nantong, China). The real-time PCR system (QuantStudio 7 Flex) was obtained from Thermo Fisher Scientific (Waltham, MA, USA). RNA extraction, reverse transcription, and qPCR kits were purchased from TransGen Biotech Co., Ltd. (Beijing, China). Antibodies against Aβ_40_ (#12990), Aβ_42_ (#14974T), and total Aβ (#8243S) were obtained from Cell Signaling Technology (Danvers, MA, USA). The anti-Aβ_17–24_ antibody (4G8, #800701) was purchased from BioLegend (San Diego, CA, USA).

### 2.3. Construction of Recombinant AAV Vectors

Recombinant AAV vectors were constructed to express human APP carrying Swedish (K670N/M671L), London (V717I), and Austrian (T714I) mutations. The AAV plasmid containing the NanoLuc reporter gene was used as the backbone, and the viral capsid was based on the AAV9 serotype. AAV9 was selected because of its broad use in nervous system gene delivery, efficient neuronal transduction, and relatively stable long-term expression after stereotaxic administration [13,15]. The hAPP-SLA sequence was inserted upstream of NanoLuc and linked by a P2A peptide. The control vector contained NanoLuc but no APP sequence.

### 2.4. Stereotaxic Injection and In Vivo Imaging

All surgical instruments were sterilized before use. Procedures involving viral vectors were performed in a Class II biosafety cabinet(BSC-1804 II A2; AIRTECH, Suzhou, China). Animals were fasted for 6 h before surgery. Anesthesia was induced with 4% isoflurane(RWD Life Science Co., Ltd., Shenzhen, China) and maintained at 1.5–2% isoflurane. Animals were fixed in a stereotaxic frame. The skull was exposed, and bregma and lambda were aligned.

CA1-based stereotaxic coordinates were selected as the intended injection site because of the important role of the CA1 region in learning and memory and its relevance to AD-related hippocampal dysfunction. The coordinates were determined according to a published stereotaxic brain atlas of the Chinese tree shrew and optimized by preliminary dye-injection experiments (Supplementary Figure S1). The final coordinates were AP −5.36 mm, ML ±6.50 mm, and DV −11.60 mm [30]. The preliminary dye-injection experiments supported stereotaxic coordinate placement but did not establish CA1-restricted vector spread or transgene expression.

The viral suspension (1 × 10^13^ vg/mL) was slowly infused bilaterally at the hippocampal CA1 coordinates, with a volume of 4 μL per side. The injection volume was selected based on preliminary volume optimization to obtain sufficient reporter expression while avoiding unnecessary high-volume injection. The needle was retained for 10 min before slow withdrawal. After injection, the skull was sealed with dental cement (Shanghai Yuyan Scientific Instrument Co., Ltd., Shanghai, China) and the incision was sutured.

At 2 weeks, 6 weeks, 5 months, and 6 months post-injection, in vivo bioluminescence imaging was performed to monitor NanoLuc reporter signals. Furimazine (Nantong Quanyi Biotechnology Co., Ltd., Nantong, China) was administered intraperitoneally (0.23 mL per 100 g body weight, 5 mmol/L), and imaging was conducted 10–15 min after Furimazine administration.

### 2.5. Behavioral Assessments

Behavioral tests were conducted before modeling and at 6 months post-injection.

#### 2.5.1. Novel Object Recognition Test

The test consisted of habituation, training, and testing phases. Animals were habituated for 30 min and allowed to explore an empty arena for 5 min. During training, two identical objects were presented, and animals explored for 10 min. After a 1 h interval, one object was replaced with a novel object. Exploration behavior was recorded for 10 min. Exploration was defined as a nose–object distance ≤ 2 cm with orientation toward the object. The recognition index (*RI*) was calculated as:

(1) $$\mathrm{RI}\;=\;\frac{T_{novel}}{T_{novel}\;+\;T_{familiar}}\times 100\%$$

#### 2.5.2. Three-Chamber Social Interaction Test

The test consisted of two phases. In the first phase, an empty cage (*E*) and a stranger (*S1*) were placed in opposite chambers. Social preference was assessed. In the second phase, a new stranger (*S2*) was introduced. Social novelty recognition was evaluated. Interaction was defined as a nose–cage distance ≤ 2 cm or full-body entry within a 10 cm zone. The social preference index (*SPI*) was calculated as:

(2) $$\mathrm{SPI}\;=\;\frac{T_{S1\;}-\;T_{E}}{T_{S1\;}+\;T_{E}}\;\times \;100\%$$

The social novelty preference index (*SNPI*) was calculated as:

(3) $$\mathrm{SNPI}\;=\;\frac{T_{S2\;}-\;T_{S1}}{T_{S2}\;+{\;T}_{S1}}\;\times \;100\%$$

### 2.6. Tissue Collection

Blood (2 mL) was collected via bilateral femoral veins. Animals were anesthetized with urethane (20%, 0.5 mL/100 g, i.p.; Shanghai Macklin Biochemical Technology Co., Ltd., Shanghai, China). After loss of reflexes, transcardial perfusion with cold phosphate-buffered saline (PBS; Beijing Solarbio Science & Technology Co., Ltd., Beijing, China) was performed. Animals were euthanized by cervical dislocation. Brains were rapidly removed. The left hippocampus was dissected, washed, snap-frozen in liquid nitrogen, and stored at −80 °C. The right hemisphere was fixed in 4% paraformaldehyde (Servicebio, Wuhan, China) for 48 h, dehydrated, embedded in paraffin(Leica Biosystems, Nussloch, Germany), and sectioned at 4 μm.

### 2.7. Molecular Analysis

Hippocampal tissues stored at −80 °C were processed for genomic DNA extraction following the manufacturer’s protocol. The extracted DNA served as the template for PCR amplification using primers designed specifically for the human triple-mutant APP sequence, in order to verify the presence of exogenous hAPP in the tree shrew hippocampus. PCR products were separated by agarose gel electrophoresis and imaged using an iBright™ 1500 Imaging System (Invitrogen, Thermo Fisher Scientific, Waltham, MA, USA). Amplicons with the expected size were then subjected to Sanger sequencing to confirm the inserted sequence. Original unprocessed PCR gel images are provided in Supplementary Figure S2.

For transcript analysis, total RNA was isolated from hippocampal tissues using an RNA extraction kit (TransGen Biotech Co., Ltd., Beijing, China), and RNA concentration and purity were assessed before reverse transcription. Qualified RNA samples were reverse-transcribed into cDNA according to the manufacturer’s instructions. RT-qPCR was performed on a real-time PCR system. GAPDH and HPRT1 were used as reference genes, and the mean Ct value of GAPDH and HPRT1 was used to calculate ΔCt values for normalization. The relative expression levels of human hAPP and endogenous tree shrew APP were analyzed, and amplification specificity was confirmed by melting curve analysis. Relative gene expression was calculated using the 2^−ΔΔCt^ method. Primer sequences and amplicon sizes are provided in Supplementary Table S1, whereas amplification efficiencies and standard-curve R^2^ values are provided in Supplementary Table S2. Representative amplification and melt peak curves for GAPDH and HPRT1 are provided in Supplementary Figure S3.

Western blotting was used to evaluate Aβ-related protein changes in hippocampal tissues. Frozen hippocampal tissues were homogenized on ice in RIPA lysis buffer (Beyotime Biotechnology, Shanghai, China; P0013) supplemented with PMSF (Beyotime Biotechnology, Shanghai, China; P1008). The supernatants were collected for protein quantification using a BCA Protein Assay Kit (Beyotime Biotechnology, Shanghai, China; P0012). Equal amounts of protein (40 μg per lane) were separated using 16.5% Tris–Tricine precast gels (Mini-PROTEAN^®^, Bio-Rad, Hercules, CA, USA; #4563066) in 1× Tris–Tricine running buffer at a constant voltage of 100 V for 100 min. Proteins were transferred to 0.22 μm PVDF membranes (Bio-Rad Laboratories, Hercules, CA, USA; L1620177) by semi-dry transfer at 300 mA for 30 min under cold conditions. The transfer buffer contained 20% methanol(ASTOON, Shanghai, China). These conditions were used to improve the separation and transfer efficiency of low-molecular-weight Aβ-immunoreactive species. After blocking with 5% non-fat milk in TBST for 1 h at room temperature, membranes were incubated overnight at 4 °C with primary antibodies against Aβ_40_ (Cell Signaling Technology, Danvers, MA, USA; #12990; 1:1000), Aβ_42_ (Cell Signaling Technology; #14974T; 1:1000), 4G8 (BioLegend, San Diego, CA, USA; #800701; 1:500), total Aβ (Cell Signaling Technology; #8243S; 1:1000), and β-actin (Affinity Biosciences, Cincinnati, OH, USA; #AF7018; 1:5000). After washing, membranes were incubated for 1 h at room temperature with HRP-conjugated goat anti-mouse IgG (Servicebio, Wuhan, China; #G1214; 1:10,000) or goat anti-rabbit IgG (Servicebio, Wuhan, China; #G1213; 1:1000), as appropriate. Protein bands were visualized using an enhanced chemiluminescence detection system and quantified using ImageJ software(version 1.54f; National Institutes of Health, Bethesda, MD, USA). Target protein levels were normalized to the corresponding β-actin signal obtained from the same membrane.

Serum Aβ_1–40_ and Aβ_1–42_ immunoreactivity was assessed using commercial rat Aβ_1–40_ and Aβ_1–42_ ELISA kits (JL10226 and JL10958, respectively; Jianglai Biotechnology Co., Ltd., Shanghai, China), according to the manufacturer’s instructions. Both kits had a detection range of 15.62–1000 pg/mL, with reported sensitivities of 4.62 pg/mL for Aβ_1–40_ and 6.11 pg/mL for Aβ_1–42_. The manufacturer-reported intra-assay and inter-assay coefficients of variation for both kits were <10%. Serum samples were diluted 1:2 and analyzed in triplicate. After incubation, washing, color development, and reaction termination, absorbance was measured at 450 nm. Standard curves were generated using kit standards at concentrations of 15.62, 31.25, 62.5, 125, 250, 500, and 1000 pg/mL, with a blank control included. Because these rat-specific ELISA kits were not independently validated in tree shrew serum for matrix effects, spike recovery, dilution parallelism, or relative recognition of endogenous tree shrew-derived versus human APP-derived Aβ species, the results were interpreted as exploratory ELISA-detected Aβ_1–40_ and Aβ_1–42_ immunoreactivity rather than absolute quantitative serum Aβ concentrations. The ELISA-derived Aβ_42_/Aβ_40_ immunoreactivity ratio was used as a supportive exploratory index of Aβ-related alterations.

### 2.8. Histopathology and Immunohistochemistry

Paraffin-embedded brain sections (4 μm) were deparaffinized, rehydrated, and subjected to hematoxylin–eosin (H&E), Nissl, Thioflavin S, and immunohistochemical staining. H&E staining was used to evaluate general hippocampal morphology. For Nissl staining, paraffin-embedded hippocampal sections were deparaffinized in an eco-friendly dewaxing and clearing solution (Servicebio, Wuhan, China; G1128) twice for 15 min each, followed by rehydration in absolute ethanol twice for 5 min each and 75% ethanol (Hunan Guangshengyuan Pharmaceutical Technology Co., Ltd., Hengyang, China) for 5 min. After rinsing in tap water, the sections were immersed in Nissl staining solution (Servicebio, Wuhan, China; G1036) for 2–5 min. The sections were then rinsed with water and briefly differentiated in 0.1% glacial acetic acid (Sinopharm Chemical Reagent Co., Ltd., Shanghai, China; #10000218). The degree of differentiation was monitored microscopically and terminated by tap-water rinsing when an appropriate contrast between Nissl substance and the background was achieved. The sections were oven-dried, cleared in xylene for 10 min, and mounted with neutral resin. Nissl-stained sections were examined and imaged using a Nikon E100 bright-field microscope (Nikon Corporation, Tokyo, Japan). Hippocampal cytoarchitecture, neuronal arrangement, cellular morphology, and the distribution of Nissl substance in the CA1 region were evaluated qualitatively. Nissl staining was used as a morphological assessment of neuronal integrity and cytoarchitecture and was not used as an independent quantitative measure of neuronal loss. For Thioflavin S staining, sections were incubated with 0.3% Thioflavin S prepared in 50% ethanol (Shanghai Yuanye Bio-Technology Co., Ltd., Shanghai, China; S19293) for 8 min, briefly differentiated in 80% ethanol, counterstained with DAPI (Servicebio, Wuhan, China; G1012), and mounted with anti-fade mounting medium (Servicebio, Wuhan, China; G1401). Fluorescence images were acquired using a Nikon Eclipse C1 microscope (Nikon Corporation, Tokyo, Japan). DAPI signals were collected using an excitation/emission wavelength range of 330–380/420 nm, whereas Thioflavin S fluorescence was acquired in the green channel using an excitation/emission wavelength range of 465–495/515–555 nm. Identical acquisition settings were applied to all groups for quantitative analysis.

For DAB-based immunohistochemistry, antigen retrieval was performed in citrate buffer (pH 6.0; Servicebio, Wuhan, China) for 4G8 and GFAP staining and in Tris–EDTA buffer (pH 8.0; Servicebio, Wuhan, China) for p-Tau (Ser202/Thr205), Synaptophysin, PSD-95, and Iba-1 staining. Sections were incubated overnight at 4 °C with primary antibodies against 4G8 (BioLegend, San Diego, CA, USA; 800701; 1:200), p-Tau (Ser202/Thr205) (Servicebio, Wuhan, China; GB113883; 1:1000), GFAP (Servicebio, Wuhan, China; GB11096; 1:2000), Iba-1 (Servicebio, Wuhan, China; GB15105; 1:500), Synaptophysin (Servicebio, Wuhan, China; GB15814; 1:1000), and PSD-95 (Servicebio, Wuhan, China; GB150080; 1:100), followed by incubation with HRP-conjugated secondary antibodies and DAB visualization. Bright-field images were acquired using a Nikon E100 microscope. For each animal, three non-overlapping fields from anatomically comparable hippocampal regions were analyzed using ImageJ software, and the mean value of the three fields was used for statistical analysis.

### 2.9. Statistical Analysis

**Figure 1:**
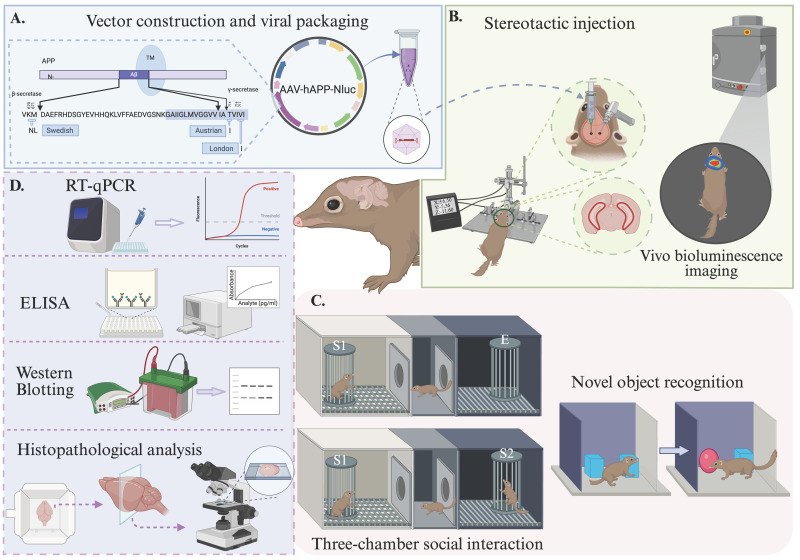
Overall experimental workflow. (**A**) Construction and packaging of the control rAAV-NLuc and experimental rAAV-hAPP-SLA-NLuc vectors. The experimental vector carries human APP harboring the Swedish, London, and Austrian mutations and is linked to NLuc through a P2A peptide. (**B**) Bilateral hippocampal stereotaxic injection and longitudinal in vivo bioluminescence imaging. Pseudocolor signals in the imaging schematic indicate relative bioluminescence intensity. (**C**) Behavioral assessments, including the three-chamber social interaction test and novel object recognition test. E denotes the empty cage, and S1 and S2 denote stranger 1 and stranger 2, respectively. In the novel object recognition schematic, the blue and red objects denote the familiar and novel objects, respectively. (**D**) Terminal molecular and histopathological analyses, including RT-qPCR, ELISA, Western blotting, and histopathological analysis. Solid and dashed arrows/connectors indicate the experimental sequence or relationships between procedures. The blue, green, pink, and purple shaded areas delineate the vector construction and packaging, stereotaxic injection and imaging, behavioral testing, and terminal molecular and histopathological analysis modules, respectively.

Normality was assessed using the Shapiro–Wilk test, and homogeneity of variance was evaluated using Levene’s test. For comparisons among the NC, VC, EXP, and AGED groups, datasets meeting both assumptions were analyzed by one-way analysis of variance followed by Tukey’s or Dunnett’s multiple-comparison test, as appropriate. Datasets meeting the normality assumption but not the homogeneity-of-variance assumption were analyzed using Welch’s ANOVA followed by Games–Howell multiple-comparison test. Datasets that did not meet the normality assumption were analyzed using the Kruskal–Wallis test followed by Dunn’s multiple-comparison test. For the three-chamber social interaction test, interaction time with the two stimuli was analyzed by two-way repeated-measures ANOVA followed by Sidak’s multiple-comparison test. The statistical test applied to each outcome is specified in the corresponding figure legend. All tests were two-sided, and *p* < 0.05 was considered statistically significant. Each data point represents one animal. Effect-size estimates for the primary behavioral and histopathological outcomes are provided in Supplementary Tables S3 and S4. The overall experimental workflow is shown in Figure 1.

## 3. Results

### 3.1. Construction and Validation of Recombinant AAV Vectors

**Figure 2:**
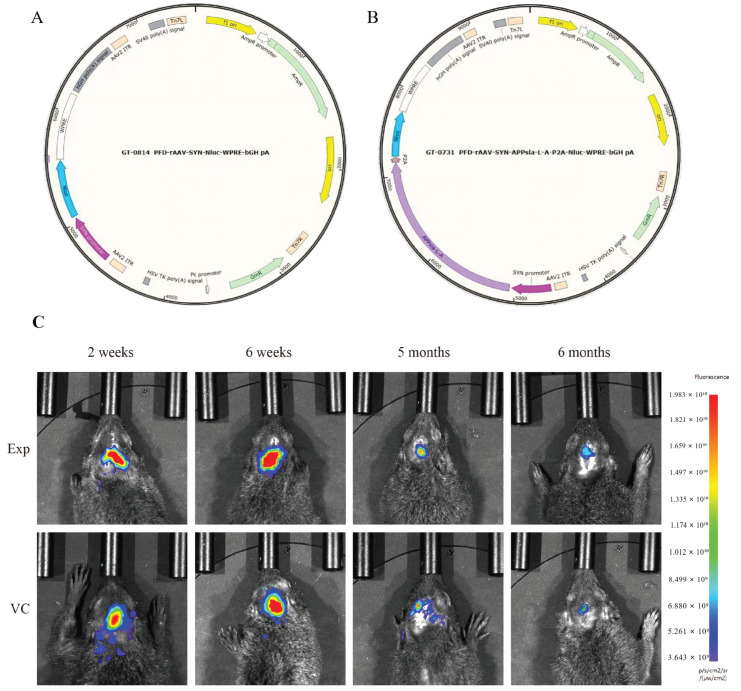
Recombinant AAV vector design and longitudinal in vivo bioluminescence monitoring in tree shrews. (**A**) Plasmid map of the control rAAV-NLuc vector. (**B**) Plasmid map of the experimental rAAV-hAPP-SLA-NLuc vector carrying human APP with Swedish, London, and Austrian mutations. (**C**) Representative in vivo bioluminescence images showing NanoLuc-related signals in the EXP and VC groups at 2 weeks, 6 weeks, 5 months, and 6 months after injection. Pseudo-color overlays represent relative bioluminescence intensity according to the corresponding color scale, with warmer colors indicating higher signal intensity and cooler colors indicating lower signal intensity.

To establish an AD-like model in the tree shrew hippocampus, a control vector and a target vector carrying human triple-mutant APP were generated. The control vector contained the Syn promoter, the NanoLuc reporter gene, WPRE, and the bGH polyA signal sequence (Figure 2A). The target vector was constructed using the same backbone. The human APP sequence carrying the Swedish, London, and Austrian mutations was inserted upstream of NanoLuc and linked through a P2A peptide. Thus, hAPP-SLA and NanoLuc were expressed under the control of the same promoter (Figure 2B). Except for the hAPP-SLA sequence, the major expression elements were identical between the control and target vectors.

To monitor viral expression in vivo, bioluminescence imaging was performed at 2 weeks, 6 weeks, 5 months, and 6 months after injection. In the EXP group, clear NanoLuc-related bioluminescence signals were detected in the cranial injection region at 2 weeks after injection. The signal remained evident at 6 weeks. Detectable NanoLuc-related signals were still observed in the cranial injection-associated region at 5 and 6 months (Figure 2C). NanoLuc-related signals were also detected in the VC group, indicating reporter gene expression in the injection region. These results showed persistent vector-associated NanoLuc reporter activity in the tree shrew brain for at least 6 months after injection. However, the in vivo bioluminescence signal did not provide sufficient spatial resolution to define the anatomical distribution of transgene expression.

### 3.2. Behavioral Alterations in AAV-hAPP-SLA-Injected Tree Shrews

Behavioral baseline testing performed before modeling showed no significant differences among the NC, VC, and EXP groups in the novel object recognition test or the three-chamber social interaction test (all *p* > 0.05).

**Figure 3:**
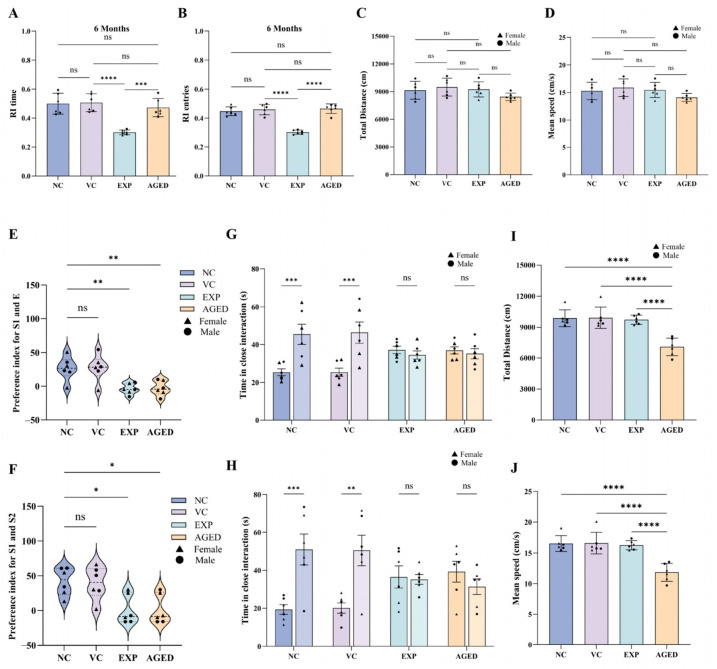
Behavioral performance of tree shrews at 6 months after AAV-hAPP-SLA injection, with individual data points stratified by sex. (**A**) Recognition index based on exploration time in the novel object recognition test. (**B**) Recognition index based on entries in the novel object recognition test. (**C**) Total distance traveled during the novel object recognition test. (**D**) Mean speed during the novel object recognition test. (**E**) Preference index for stranger 1 versus the empty cage during the social approach phase. (**F**) Preference index for stranger 2 versus stranger 1 during the social novelty phase. (**G**) Close interaction time during the social approach phase. (**H**) Close interaction time during the social novelty phase. (**I**) Total distance traveled during the three-chamber social test. (**J**) Mean speed during the three-chamber social test. Data are presented as mean ± SEM, with individual data points shown. For panels (**A**–**F**,**I**,**J**), pooled group comparisons were performed using one-way ANOVA followed by Tukey’s post hoc test. For panels (**G**,**H**), close interaction time was analyzed using two-way repeated-measures ANOVA, with group as the between-subject factor and stimulus as the within-subject factor, followed by Sidak’s multiple-comparison test. Triangles indicate females and circles indicate males. ns, not significant; * *p* < 0.05; ** *p* < 0.01; *** *p* < 0.001; **** *p* < 0.0001. NC, negative control group; VC, vector control group; EXP, experimental group; AGED, aged group.

At 6 months after injection, the recognition index based on exploration time was significantly lower in the EXP group than in the NC and VC groups (both *p* < 0.0001) and the AGED group (*p* < 0.001). No significant differences were observed among the NC, VC, and AGED groups (Figure 3A). Similarly, the recognition index based on entries was significantly reduced in the EXP group compared with the NC, VC, and AGED groups (all *p* < 0.0001), whereas no significant differences were detected among the NC, VC, and AGED groups (Figure 3B).

No significant differences in total distance traveled or mean speed were observed among the four groups during the novel object recognition test (Figure 3C,D).

During the social approach phase, no significant difference in the S1/E preference index was observed between the NC and VC groups. However, the EXP and AGED groups showed lower S1/E preference indices than the NC group (both *p* < 0.01; Figure 3E). During the social novelty phase, the S2/S1 preference index was also reduced in the EXP and AGED groups compared with the NC group (both *p* < 0.05), while no significant difference was detected between the NC and VC groups (Figure 3F).

Analysis of close interaction time showed that the NC and VC groups spent significantly more time interacting with stranger 1 than with the empty cage during the social approach phase (both *p* < 0.001). In contrast, no significant difference was detected between stranger 1 and the empty cage in the EXP or AGED group (Figure 3G). During the social novelty phase, the NC and VC groups spent significantly more time interacting with the novel stranger 2 than with the familiar stranger 1 (*p* < 0.001 and *p* < 0.01, respectively), whereas no significant difference was observed in the EXP or AGED group (Figure 3H).

During the three-chamber test, total distance traveled and mean speed were significantly lower in the AGED group than in the NC, VC, and EXP groups (all *p* < 0.0001; Figure 3I,J).

Overall, the EXP group exhibited impaired novel object recognition, reduced social approach preference, and impaired social novelty recognition at 6 months after AAV-hAPP-SLA injection. The AGED group showed no significant impairment in novel object recognition but showed reduced social approach and social novelty preference.

### 3.3. Verification of hAPP Expression and Aβ-Related Molecular Alterations

**Figure 4:**
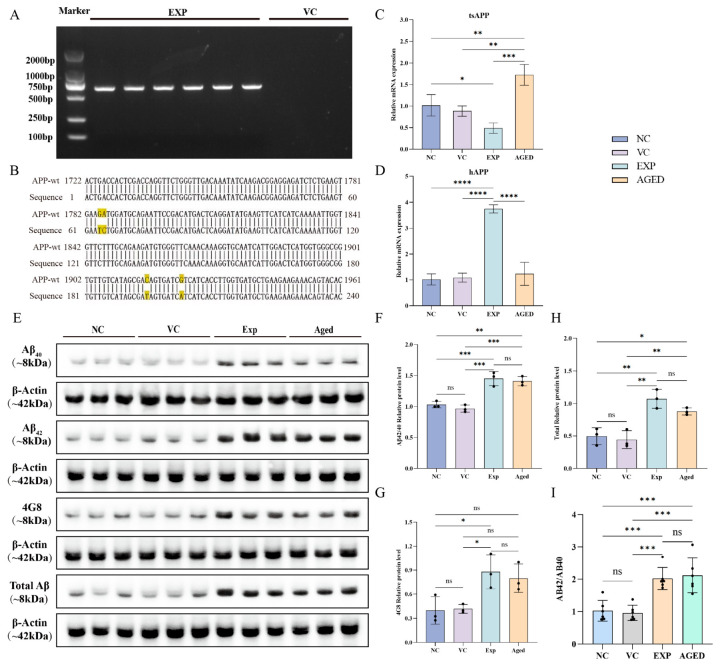
Hippocampal hAPP expression and Aβ-related molecular alterations after AAV-hAPP-SLA injection. (**A**) PCR detection of the exogenous hAPP fragment in brain tissues from the EXP and VC groups. (**B**) Sanger sequencing alignment confirming the expected mutation sites in the amplified hAPP fragment. Highlighted nucleotides indicate the Swedish, Austrian, and London mutation sites. (**C**) Relative expression of endogenous tree shrew APP (tsAPP) in hippocampal tissues. (**D**) Relative expression of exogenous human APP (hAPP) in hippocampal tissues. RT-qPCR data were normalized using GAPDH and HPRT1 as reference genes. (**E**) Representative Western blot bands of Aβ_40_, Aβ_42_, 4G8-reactive APP/Aβ-related signal, and total Aβ, with β-actin used as the loading control. The original images can be found in the Supplementary Materials. (**F**) Aβ_42_/Aβ_40_ relative protein ratio based on Western blot analysis. (**G**) Relative 4G8-reactive APP/Aβ-related protein level. (**H**) Relative total Aβ protein level. (**I**) ELISA-derived Aβ_42_/Aβ_40_ immunoreactivity ratio in tree shrew serum. Data are presented as mean ± SEM, with individual data points shown. Group comparisons were performed using one-way ANOVA followed by Tukey’s post hoc test. ns, not significant; * *p* < 0.05; ** *p* < 0.01; *** *p* < 0.001; **** *p* < 0.0001. NC, negative control group; VC, vector control group; EXP, experimental group; AGED, aged group.

To verify the introduction of exogenous hAPP after AAV-hAPP-SLA injection, PCR amplification was performed using hippocampal tissue samples. Agarose gel electrophoresis showed clear target bands in all EXP samples, whereas no corresponding bands were detected in the VC group. This result indicated successful introduction of the exogenous hAPP fragment into the hippocampal tissue of the EXP group (Figure 4A). Sanger sequencing alignment further showed that the amplified products from the EXP group matched the designed sequence and contained the expected Swedish, Austrian, and London mutation sites, confirming the identity of the introduced hAPP-SLA fragment (Figure 4B).

After normalization using GAPDH and HPRT1 as reference genes, RT-qPCR analysis revealed distinct expression patterns of endogenous tree shrew APP (tsAPP) and exogenous human APP (hAPP) among groups. tsAPP expression was lower in the EXP group than in the NC and VC groups, whereas the AGED group showed higher tsAPP expression than the other groups (Figure 4C). In contrast, hAPP expression was markedly increased in the EXP group but remained at low levels in the NC, VC, and AGED groups (Figure 4D). These findings confirmed that AAV-hAPP-SLA injection induced exogenous hAPP expression in the hippocampal tissue of EXP tree shrews. The EXP and AGED groups showed distinct APP transcriptional profiles, characterized by increased exogenous hAPP expression in the EXP group and higher endogenous tsAPP expression in the AGED group.

Western blotting detected Aβ-immunoreactive signals migrating predominantly at approximately 8 kDa in hippocampal lysates. These signals were more prominent in the EXP group than in the NC and VC groups, and enhanced signals were also observed in the AGED group (Figure 4E). Densitometric analysis showed that the relative Aβ_42_ protein level, 4G8-reactive APP/Aβ-related signal, and total Aβ immunoreactivity were increased to different extents in the EXP and AGED groups (Figure 4F–H). The approximately 8 kDa signals are described as Aβ-immunoreactive species and should not be interpreted as definitively identified monomeric Aβ. Because synthetic Aβ peptide standards were not run alongside tissue samples, the precise molecular identity of these immunoreactive bands could not be conclusively determined. These findings indicate that AAV-hAPP-SLA injection was associated with hippocampal Aβ-related molecular alterations, while the older-aged tree shrews also showed Aβ-related changes. The corresponding original uncropped Western blot images are provided in Supplementary Figure S4.

ELISA analysis showed no significant difference in the ELISA-derived Aβ_42_/Aβ_40_ immunoreactivity ratio between the NC and VC groups. Compared with the NC and VC groups, the EXP and AGED groups showed significantly increased ELISA-derived Aβ_42_/Aβ_40_ immunoreactivity ratios. No significant difference was detected between the EXP and AGED groups (Figure 4I). Because the rat-specific kits were not validated in tree shrew serum, these findings were interpreted as exploratory supportive evidence of altered serum Aβ-related immunoreactivity rather than as absolute quantitative measurements of Aβ concentrations. In addition, the assay could not distinguish human APP-derived Aβ from endogenous tree shrew-derived Aβ species in the EXP group.

Overall, AAV-hAPP-SLA injection induced exogenous hAPP expression in the tree shrew hippocampus and was accompanied by an increased ELISA-derived Aβ_42_/Aβ_40_ immunoreactivity ratio, enhanced 4G8-reactive Aβ signal, and elevated total Aβ levels. Aβ-related alterations were observed in both the EXP and AGED groups, but the source and expression pattern of APP differed between the two groups.

### 3.4. Histopathological Changes in the Hippocampus

**Figure 5:**
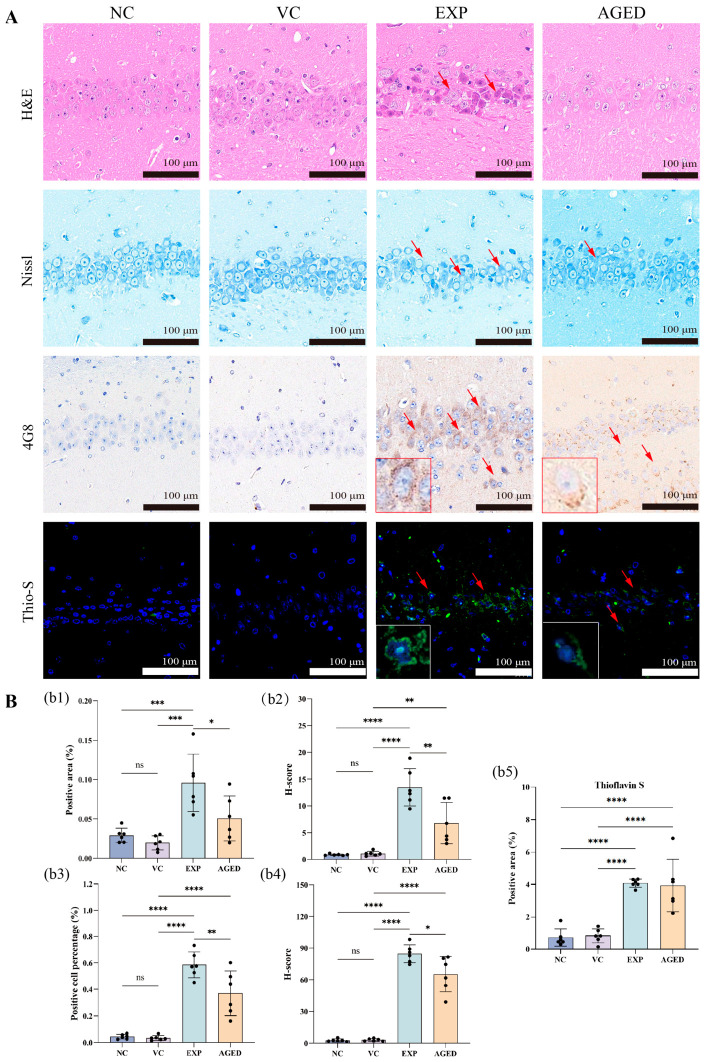
Hippocampal histopathological changes and 4G8 immunoreactivity and Thioflavin S-positive staining after AAV-hAPP-SLA injection. (**A**) Representative hippocampal sections from the NC, VC, EXP, and AGED groups stained with hematoxylin–eosin (H&E), Nissl staining, 4G8 immunohistochemistry, and Thioflavin S staining. Red arrows indicate representative pathological alterations or positive signals. Red boxes delineate regions shown at higher magnification in the corresponding insets. 4G8-immunoreactive signals are shown in brown, whereas Thioflavin S-positive signals and nuclei are shown in green and blue, respectively. Different bar colors in panel B indicate the four study groups, as labeled on the x-axis. Scale bars = 100 μm. (**B**) Quantitative analysis of 4G8 immunoreactivity and Thioflavin S-positive area. (**b1**) 4G8-positive area. (**b2**) H-score based on positive area. (**b3**) Percentage of 4G8-positive cells. (**b4**) H-score based on positive cells. (**b5**) Thioflavin S-positive area. Data are presented as mean ± SEM, with individual data points shown. Group comparisons were performed using one-way ANOVA followed by Tukey’s post hoc test. ns, not significant; * *p* < 0.05; ** *p* < 0.01; *** *p* < 0.001; **** *p* < 0.0001. NC, negative control group; VC, vector control group; EXP, experimental group; AGED, aged group.

To further evaluate histopathological changes after AAV-hAPP-SLA injection, hippocampal tissues were examined using H&E staining, Nissl staining, 4G8 immunohistochemistry, and Thioflavin S staining. H&E staining showed relatively regular cellular arrangement and clear cellular layers in the hippocampus of the NC and VC groups. No obvious structural disruption was observed. In contrast, focal loosening of cellular arrangement and local cytoarchitectural alterations were observed in the EXP group. The AGED group also showed reduced cellular compactness and less organized tissue architecture. Nissl staining showed preserved neuronal morphology and clear Nissl body staining in the NC and VC groups. Reduced Nissl substance staining and less regular neuronal arrangement were qualitatively observed in both the EXP and AGED groups (Figure 5A).

4G8 immunohistochemistry showed only weak 4G8-positive signals in the hippocampus of the NC and VC groups. In the EXP group, 4G8-reactive APP/Aβ-related immunoreactivity was markedly increased and appeared as strong brown immunoreactive signals. Increased 4G8-positive signals were also observed in the AGED group, although the overall signal was less prominent than that in the EXP group. Because the 4G8 epitope is conserved across species, the detected signal is described as 4G8-reactive APP/Aβ-related immunoreactivity and does not distinguish exogenous human from endogenous tree shrew APP/Aβ-derived species.

Thioflavin S staining showed few fluorescent signals in the NC and VC groups. In contrast, Thioflavin S-reactive signals were increased in both the EXP and AGED groups, with the EXP group showing the most prominent overall signal (Figure 5A). At higher magnification, the positive signals appeared predominantly diffuse, granular, punctate, and non-compact, rather than consistently forming compact, sharply demarcated plaque-like structures. Because Thioflavin S staining alone cannot identify the molecular composition or determine the intracellular versus extracellular localization of the labeled structures, these signals were described as Thioflavin S-reactive aggregate-associated signals.

To quantify 4G8 immunohistochemistry, the positive area, positive area H-score, positive cell percentage, and positive cell H-score were analyzed. No significant differences were observed between the NC and VC groups for any parameter. Compared with the NC and VC groups, the EXP group showed a significantly increased 4G8-positive area. The AGED group also showed an increased positive area. The positive area was higher in the EXP group than in the AGED group (Figure 5(Bb1)). Positive area H-score analysis showed that the EXP group was significantly higher than the NC, VC, and AGED groups. The AGED group was also higher than the control groups (Figure 5(Bb2)).

The positive cell percentage showed a similar pattern. The EXP group showed a significantly higher percentage of 4G8-positive cells than the NC and VC groups. The AGED group also showed an increase compared with the control groups, whereas the EXP group showed the highest mean value (Figure 5(Bb3)). Positive cell H-score analysis further showed that both the EXP and AGED groups were higher than the NC and VC groups, with the highest level observed in the EXP group (Figure 5(Bb4)). Quantification of the Thioflavin S-reactive fluorescence-positive area further showed increased aggregate-associated signal area in the EXP and AGED groups compared with the NC and VC groups, with the highest mean value observed in the EXP group (Figure 5(Bb5)).

Taken together, at 6 months after AAV-hAPP-SLA injection, the EXP group showed neuronal morphological alterations, reduced Nissl staining, increased 4G8-reactive APP/Aβ-related immunoreactivity, and enhanced Thioflavin S-positive signals in the hippocampus. The AGED group also showed overlapping histopathological and amyloid-related changes, whereas the EXP group generally exhibited a greater 4G8-related pathological burden.

### 3.5. Increased AT8 (Ser202/Thr205) Immunoreactivity and Glial Reactivity in the Hippocampus

**Figure 6:**
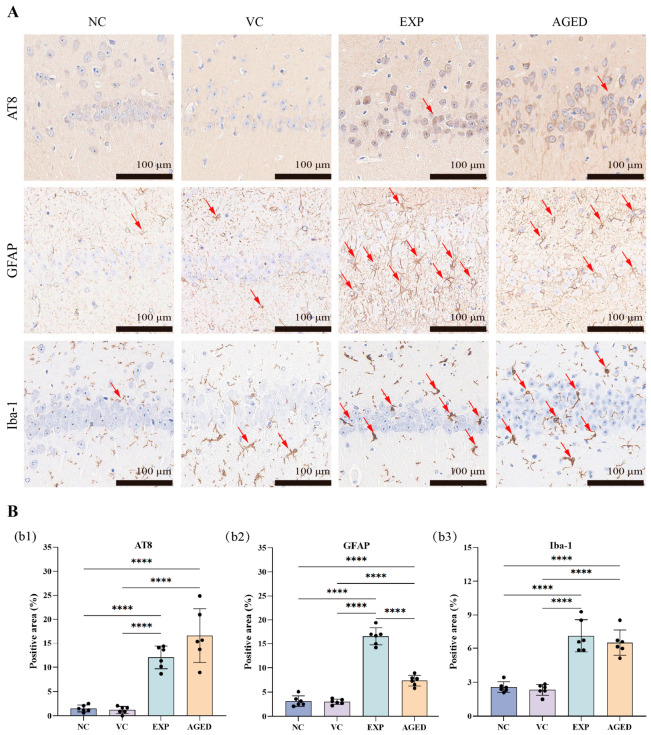
Hippocampal AT8 (Ser202/Thr205) immunoreactivity and glial reactivity after AAV-hAPP-SLA injection. (**A**) Representative immunohistochemical staining of AT8 (Ser202/Thr205), GFAP, and Iba-1 in hippocampal sections from the NC, VC, EXP, and AGED groups. Red arrows indicate representative positive signals. Scale bars = 100 μm. (**B**) Quantitative analysis of the positive area of (**b1**) AT8 (Ser202/Thr205), (**b2**) GFAP, and (**b3**) Iba-1. Data are presented as mean ± SEM, with individual data points shown. Each dot represents one animal. Group comparisons were performed using one-way ANOVA followed by Tukey’s post hoc test. **** *p* < 0.0001. Different bar colors indicate the four study groups, as labeled on the x-axis.NC, negative control group; VC, vector control group; EXP, experimental group; AGED, aged group.

Immunohistochemical staining showed weak AT8 (Ser202/Thr205) immunoreactivity in the hippocampus of the NC and VC groups. In contrast, more prominent AT8-positive signals were observed in both the EXP and AGED groups (Figure 6A). Quantitative analysis showed that the AT8-positive area was significantly increased in the EXP and AGED groups compared with the NC and VC groups (Figure 6(Bb1)).

GFAP immunoreactivity was relatively limited in the NC and VC groups but was increased in the EXP and AGED groups. Quantitative analysis showed that the GFAP-positive area was significantly higher in both the EXP and AGED groups than in the NC and VC groups, with the EXP group showing a higher level than the AGED group (Figure 6(Bb2)). Similarly, Iba-1-positive signals were increased in the EXP and AGED groups compared with the NC and VC groups, and the Iba-1-positive area was significantly increased in both groups (Figure 6A,Bb3).

Collectively, these findings indicate that AAV-hAPP-SLA injection was associated with increased hippocampal AT8 (Ser202/Thr205) immunoreactivity and enhanced astrocytic and microglial reactivity at 6 months after injection. The AGED group showed partially overlapping changes.

### 3.6. Altered Synaptic Marker Immunoreactivity in the Hippocampus

**Figure 7:**
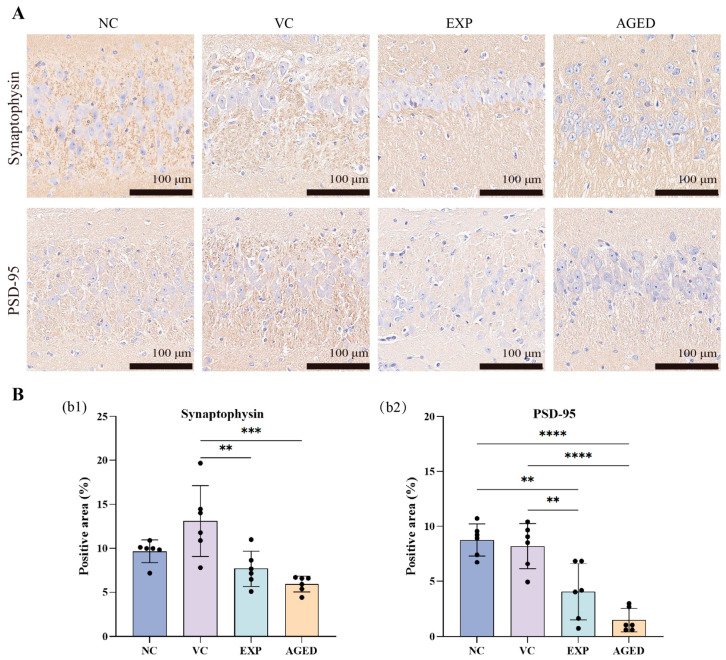
Altered hippocampal synaptic marker immunoreactivity after AAV-hAPP-SLA injection. (**A**) Representative immunohistochemical staining of Synaptophysin and PSD-95 in hippocampal sections from the NC, VC, EXP, and AGED groups. Scale bars = 100 μm. (**B**) Quantitative analysis of the positive area of (**b1**) Synaptophysin and (**b2**) PSD-95. Data are presented as mean ± SEM, with individual data points shown. Each dot represents one animal. Group comparisons were performed using one-way ANOVA followed by Tukey’s post hoc test. ** *p* < 0.01; *** *p* < 0.001; **** *p* < 0.0001. NC, negative control group; VC, vector control group; EXP, experimental group; AGED, aged group.

Synaptophysin and PSD-95 immunoreactivities were detected in the hippocampus of all groups. Synaptophysin staining appeared weaker in the EXP and AGED groups than in the VC group (Figure 7A). Quantitative analysis showed that the Synaptophysin-positive area was significantly lower in the EXP and AGED groups than in the VC group. In addition, the AGED group showed a significantly lower Synaptophysin-positive area than the NC group (*p* = 0.0497), whereas the EXP group did not differ significantly from the NC group (*p* = 0.4786). No significant difference was observed between the NC and VC groups (*p* = 0.0921) (Figure 7(Bb1)).

For PSD-95, the positive area was significantly reduced in both the EXP and AGED groups compared with the NC and VC groups (Figure 7(Bb2)). These findings indicate altered presynaptic and postsynaptic marker immunoreactivity in the hippocampus of EXP tree shrews, with similar reductions in the AGED group.

## 4. Discussion

In this study, AAV-mediated delivery of human triple-mutant APP following bilateral stereotaxic injection directed at CA1 coordinates induced Aβ-related molecular, pathological, and behavioral alterations in tree shrews. This phenotype was supported by persistent vector-associated reporter signals, terminal detection of exogenous hAPP expression, Aβ-related molecular changes, 4G8-reactive APP/Aβ-related immunoreactivity, Thioflavin S-positive amyloid-like aggregation, AT8 (Ser202/Thr205) immunoreactivity, glial reactivity, altered synaptic marker immunoreactivity, and behavioral abnormalities in novel object recognition and social interaction. These findings support the establishment of a human APP-driven, Aβ-related AD-like phenotype in tree shrews, while this model should not be interpreted as a complete recapitulation of the full pathological course of human AD.

First, the present study confirmed that AAV-mediated hAPP-SLA expression could be maintained in the tree shrew brain for at least 6 months. In vivo bioluminescence imaging showed that NanoLuc-related signals were detected in the EXP group as early as 2 weeks after injection and remained observable in the cranial injection-associated region at 6 months [15]. PCR amplification and Sanger sequencing confirmed the presence and identity of the exogenous hAPP-SLA fragment in hippocampal tissue, whereas RT-qPCR further demonstrated hAPP transcript expression at the 6-month endpoint. Because in vivo bioluminescence imaging does not provide cellular-resolution mapping of viral transduction, these signals should be interpreted as evidence of persistent vector-associated reporter activity rather than proof of CA1-restricted expression. Abnormal Aβ deposition is one of the core pathological hallmarks of AD, and aberrant Aβ generation is closely associated with amyloidogenic APP processing mediated by β-secretase and γ-secretase [31,32]. Previous studies in tree shrews have shown that hippocampal injection of Aβ_1–40_ or Aβ_25–35_ can induce cognitive impairment, neuronal apoptosis, and related molecular changes [25,26]. Unlike direct injection of exogenous Aβ peptides, the hAPP-SLA strategy used in this study provides a sustained human APP-related pathogenic trigger, which may facilitate investigation of the relationship among APP expression, Aβ-related molecular alterations, histopathological responses, and behavioral abnormalities over time [33].

RT-qPCR results showed that the EXP group was mainly characterized by a marked increase in exogenous hAPP expression, whereas the AGED group was characterized by increased endogenous tsAPP expression. This finding should be interpreted as a difference in APP transcriptional patterns rather than direct evidence for distinct regulatory mechanisms. In particular, the decreased tsAPP expression in the EXP group may reflect a compensatory transcriptional response to exogenous hAPP expression or Aβ-related stress; however, altered cellular composition of bulk hippocampal tissue or other non-cell-autonomous effects may also contribute. The present data do not establish a causal mechanism for tsAPP downregulation. Similarly, the higher tsAPP expression in the AGED group is consistent with age-associated endogenous APP-related changes but does not define their upstream regulatory basis [34,35]. Therefore, the AAV-hAPP-SLA model is more appropriately defined as a human APP-driven Aβ-related tree shrew model with a defined molecular trigger, rather than as a direct equivalent of the phenotype observed in the older-aged reference group. Although this model reproduces selected Aβ-related molecular, pathological, and behavioral features, it does not fully recapitulate the complex and heterogeneous disease course of human AD [8,9].

Western blotting detected Aβ-immunoreactive signals migrating predominantly at approximately 8 kDa in hippocampal lysates, with more prominent signals in the EXP group and enhanced signals also observed in the AGED group. Densitometric analysis further showed increased relative Aβ_42_ protein level, 4G8-reactive APP/Aβ-related signal, and total Aβ immunoreactivity in the EXP and AGED groups. The approximately 8 kDa signals may be compatible with putative dimeric or low-order oligomeric Aβ-related species; however, their precise molecular identity and assembly state cannot be conclusively assigned from the present Western blot data alone. Synthetic Aβ_1–40_ and Aβ_1–42_ peptide standards were not included for co-migration, and therefore the detected signals should not be interpreted as definitively identified monomeric Aβ species. In addition, because the 4G8 epitope is conserved across species, the 4G8 signal should be interpreted as combined APP/Aβ-related immunoreactivity rather than as a human Aβ-specific readout. ELISA detected an increased serum Aβ_42_/Aβ_40_ immunoreactivity ratio in both the EXP and AGED groups. However, because the rat-specific ELISA kits were not independently validated in tree shrew serum for matrix effects, spike recovery, or dilution parallelism, these findings should be interpreted as exploratory supportive observations rather than as absolute quantitative measurements of serum Aβ. Moreover, the assay could not distinguish human APP-derived Aβ from endogenous tree shrew-derived Aβ species in the EXP group. Therefore, the ELISA-derived Aβ_42_/Aβ_40_ immunoreactivity ratio should not be interpreted as direct evidence of altered Aβ production, circulation, or hippocampal Aβ composition. The AGED animals were approximately 78 months old at terminal tissue collection and showed an increased ELISA-derived Aβ_42_/Aβ_40_ immunoreactivity ratio together with elevated levels of several Aβ-related proteins, indicating the presence of age-associated Aβ-related molecular alterations. In captive-bred Chinese tree shrews, reported lifespan estimates are approximately 6–8 years, although chronological aging categories may vary across lineages, breeding colonies, and husbandry conditions [36,37]. The terminal age of the AGED group was within the range used in previous aging- and neurodegeneration-related studies of tree shrews. For example, Aβ- and APP-immunoreactive structures were examined in tree shrews aged 6 years 6 months to 7 years 5 months [38], whereas hippocampal oxidative stress, Tau hyperphosphorylation, microglial alterations, and AD-like pathological changes have also been reported in older tree shrews [23,24]. Therefore, the AGED group in the present study was used as an older-aged reference group for age-associated alterations rather than as a complete representation of the natural aging trajectory or end-stage senescence.

Histopathological analysis further showed qualitative cytoarchitectural alterations and reduced Nissl staining in the hippocampus of the EXP group. These findings are consistent with neuronal structural disturbance but do not by themselves establish quantitative neuronal loss. In parallel, 4G8 immunohistochemistry showed increased 4G8-reactive APP/Aβ-related immunoreactivity in the EXP group, and quantitative analysis of positive area, area-based H-score, positive-cell percentage, and cell-based H-score confirmed a higher signal than that in the NC and VC groups. Most 4G8-related indices were also higher in the EXP group than in the AGED group [27,28,29,30]. Thioflavin S staining showed an increased positive area in the EXP and AGED groups, with the highest mean value observed in the EXP group. Morphologically, the Thioflavin S-reactive signals were predominantly diffuse, granular, punctate, and non-compact rather than consistently forming sharply demarcated plaque-like structures. Because Thioflavin S labels β-sheet-rich aggregates without identifying the protein source, these signals should be interpreted as aggregate-associated amyloid-like signals rather than definitive mature extracellular amyloid plaques [39,40]. In addition, because co-labeling with Aβ-specific antibodies, neuronal markers, or confocal imaging was not performed, the intracellular versus extracellular localization of these Thioflavin S-reactive structures could not be determined in the present study. Together, the molecular, 4G8 immunohistochemical, and Thioflavin S results support increased Aβ-related and amyloid-like pathological changes in the hippocampus after AAV-hAPP-SLA injection.

AT8 (Ser202/Thr205) recognizes Tau phosphorylated at Ser202 and Thr205 [41]. In the present study, AT8 (Ser202/Thr205) immunoreactivity was increased in the EXP and AGED groups, indicating abnormal Tau phosphorylation at this epitope. Astrocytic and microglial reactivity are recognized components of AD-associated pathology [42]. GFAP- and Iba-1-positive areas were also increased in both groups, with the EXP group showing a stronger GFAP response than the AGED group. Altered pre- and postsynaptic marker expression is also commonly observed in AD-related pathology [43]. In addition, Synaptophysin-positive area was reduced in the EXP and AGED groups compared with the VC group. The AGED group also showed a lower Synaptophysin-positive area than the NC group, whereas the EXP group did not differ significantly from the NC group. Although the NC and VC groups showed a modest numerical difference, this comparison did not reach statistical significance (Tukey-adjusted *p* = 0.0921). Because NC animals did not undergo stereotaxic surgery or vector injection, whereas VC animals underwent the control procedure and received the control vector, the numerical NC–VC difference may reflect inter-animal biological variability and/or minor procedure-related variation. However, these sources of variation were not separately examined, and the present data do not support the conclusion that surgery or control-vector administration altered Synaptophysin immunoreactivity. In contrast, PSD-95-positive area was reduced in both the EXP and AGED groups compared with the NC and VC groups. These findings indicate that AAV-hAPP-SLA injection was accompanied by Tau phosphorylation-related changes, enhanced astrocytic and microglial reactivity, and altered pre- and postsynaptic marker immunoreactivity [44,45]. Nevertheless, these data do not establish neurofibrillary tangle formation, glial-cell subtype polarization, cytokine-mediated neuroinflammation, or direct loss of synapse number or function.

Behavioral outcomes are an important component for evaluating the functional consequences of this model. In the novel object recognition test, the EXP group showed significantly reduced recognition indices based on both exploration time and entry number, whereas the AGED group did not show significant impairment in this task (Figure 3A,B). Novel object recognition depends on the ability to discriminate familiar from novel stimuli and involves coordinated cortical and hippocampal networks [46]. Tree shrews have strong visual recognition ability, and previous studies have shown that they can exhibit stable novel object recognition behavior [47,48]. Because the retention interval used in the present study was 1 h, the observed phenotype is more appropriately interpreted as impaired short-delay novel object recognition performance rather than direct evidence of long-term memory impairment. Notably, total distance traveled and mean speed did not differ among the four groups during the novel object recognition test (Figure 3C,D), indicating that the reduced recognition indices in the EXP group were not accompanied by an overt reduction in overall locomotor activity during this task. The absence of significant novel object recognition impairment in the AGED group further indicates that the EXP and AGED groups showed distinct behavioral profiles in this task.

The three-chamber social interaction test showed that both the EXP and AGED groups had reduced S1/E and S2/S1 preference indices and failed to show significant close-interaction preference for S1 over the empty cage or for S2 over S1 (Figure 3E–H). In addition to memory decline, patients with AD may exhibit neuropsychiatric and social-cognitive alterations, including reduced social engagement and impaired social cognition [49,50,51,52]. Social recognition and social novelty preference involve complex hippocampal–limbic networks, including CA2, ventral CA1, and related downstream projections [53,54]. In addition, tree shrews exhibit territoriality and stress sensitivity, and social stimuli, environmental novelty, and stress states may influence performance in social interaction tasks [55]. Notably, total distance traveled and mean speed during the three-chamber test were significantly reduced in the AGED group, whereas no comparable reduction was observed in the EXP group (Figure 3I,J). Therefore, the reduced social preference in the EXP group occurred without an overt decrease in overall locomotor activity during the task and was consistent with APP/Aβ-related alterations affecting hippocampus-associated social memory networks [56]. In contrast, although the AGED group also showed reduced social preference, this finding should be interpreted cautiously because it was accompanied by reduced locomotor activity. Age-related changes in social motivation, stress responsiveness, and limbic circuit function may contribute to this phenotype, but these possibilities were not directly examined in the present study [57].

The comparison between the EXP and AGED groups is an important component of this study. Both groups showed Aβ-related molecular and pathological alterations and displayed abnormalities in social behavioral measures. However, their behavioral profiles were not identical. The EXP group was mainly characterized by increased exogenous hAPP expression, more prominent 4G8-reactive and Thioflavin S-positive pathology, increased AT8 (Ser202/Thr205) immunoreactivity, stronger GFAP reactivity, reduced novel object recognition indices, and reduced social preference without a significant decrease in task-related locomotor activity. In contrast, the AGED group was characterized by higher endogenous tsAPP expression, age-associated APP/Aβ-related changes, reduced social preference accompanied by lower total distance traveled and mean speed during the three-chamber test, and no significant impairment in novel object recognition. These findings indicate that the AAV-hAPP-SLA model reproduced selected Aβ-related alterations that partially overlapped with aging-related changes but was not equivalent to the phenotype observed in the older-aged reference group. More precisely, this model represents a human APP-driven Aβ-related tree shrew model with a defined pathogenic trigger and a relatively short modeling period [44,45].

Tree shrews have particular value as experimental animals in AD model systems. Compared with conventional rodents, tree shrews show several features that are more relevant to primate biology and translational neuroscience, including diurnal activity, visually guided behavior, a well-developed visual system and conserved neurobiological characteristics, as well as a primate-like Aβ sequence and AD-related genetic features [17,18,19,20,21]. Compared with non-human primates, tree shrews are smaller, less costly to maintain, and more feasible for moderate-scale mechanistic and interventional studies [19]. Therefore, tree shrews may provide a useful intermediate model between rodents and non-human primates [18,19]. The present findings further indicate that AAV-mediated expression of human triple-mutant APP can establish an Aβ-related AD-like model in tree shrews, supported by molecular, pathological, and behavioral evidence. Accordingly, this human APP-driven, Aβ-related tree shrew model may serve as a complementary non-rodent platform for investigating APP/Aβ-related pathological processes and their associated behavioral consequences [58,59].

Nevertheless, no single experimental model can fully reproduce the temporal progression, pathological heterogeneity, and clinical complexity of human AD [60]. Several limitations of the present study should therefore be acknowledged. First, although viral injection was directed at CA1-based stereotaxic coordinates, the anatomical spread, cellular distribution, and transduction efficiency of the AAV vector were not directly mapped. Therefore, the present findings should not be interpreted as evidence of CA1-restricted or hippocampus-restricted transgene expression. Future studies using hAPP- or NanoLuc-based immunofluorescence mapping together with cell-type-specific markers will be required to define the precise spatial and cellular distribution of vector-mediated expression. Second, terminal molecular and histopathological analyses were performed only at 6 months after injection. Preliminary behavioral testing at 4 months did not reveal robust abnormalities, and no corresponding molecular or pathological analyses were performed at that earlier time point. In accordance with the 3R principle, particularly reduction of animal use, 6 months was selected as the principal endpoint for terminal tissue collection. Nevertheless, the absence of clear behavioral changes at 4 months does not exclude earlier molecular or pathological alterations. Future studies incorporating earlier time points are required to clarify the temporal sequence from transgene expression and Aβ-related molecular changes to tissue pathology and behavioral impairment.

Third, although the present study now includes AT8 (Ser202/Thr205), GFAP, Iba-1, Synaptophysin, and PSD-95 staining, these analyses remain marker-based histological assessments. Additional biochemical, ultrastructural, and functional studies will be required to determine whether the observed staining changes correspond to neurofibrillary pathology, specific glial phenotypes, synaptic dysfunction, or neuronal loss. Fourth, although sex was balanced during group allocation, sex-to-sample linkage was not retained in the final analytical records for all terminal molecular and histopathological specimens. Therefore, retrospective sex-stratified analyses of these endpoint data could not be performed, and the present molecular and histopathological findings should not be interpreted as sex-specific effects. Fifth, the 4G8 signal cannot distinguish exogenous human from endogenous tree shrew APP/Aβ-derived species. In addition, synthetic Aβ_1–40_ and Aβ_1–42_ peptide standards were not included in the Western blot experiments; therefore, the approximately 8 kDa Aβ-immunoreactive signals could not be validated by co-migration and should not be assigned a definitive molecular identity or assembly state. Future studies using synthetic peptide standards, human-specific assays, biochemical fractionation, and orthogonal analytical approaches will be required to define the relative contribution and molecular composition of exogenous and endogenous APP/Aβ-related species.

Finally, the AGED group represented a single older-aged cohort that was approximately 72 months old at enrollment and approximately 78 months old at terminal tissue collection. Although this terminal age is within the range reported for older captive-bred Chinese tree shrews and is comparable to the age ranges used in previous tree shrew aging and neurodegeneration studies [23,24,36,37,38], a single cross-sectional age group cannot define the temporal progression of spontaneous age-associated AD-like alterations. Future studies incorporating multiple age cohorts will be required to characterize the progression of APP/Aβ-related, Tau-related, glial, synaptic, and behavioral alterations across the aging process.

## 5. Conclusions

In this study, an Aβ-related AD-like tree shrew model was established and characterized through AAV-mediated expression of human triple-mutant APP. Following AAV-hAPP-SLA delivery, the EXP group showed exogenous hAPP expression, increased relative hippocampal Aβ_42_ protein levels, enhanced 4G8-reactive APP/Aβ-related immunoreactivity, elevated total Aβ immunoreactivity, increased ELISA-derived serum Aβ_42_/Aβ_40_ immunoreactivity ratios, and enhanced Thioflavin S-reactive aggregate-associated signals. These molecular and pathological alterations were accompanied by increased AT8 (Ser202/Thr205) immunoreactivity, enhanced glial reactivity, altered synaptic marker immunoreactivity, reduced short-delay recognition-related performance, reduced social approach preference, and reduced social novelty preference.

Although the AAV-hAPP-SLA tree shrew model did not fully recapitulate the phenotype of naturally aged tree shrews, it shared some Aβ-related molecular, pathological, and social behavioral features with the aged group. It nevertheless differed from naturally aged tree shrews in the APP transcriptional profile, the extent of 4G8-reactive and Thioflavin S-positive pathological changes, and object recognition performance. These findings suggest that the AAV-hAPP-SLA tree shrew model may serve as a human APP-driven, Aβ-related, non-rodent AD-like model for investigating APP/Aβ-associated pathological processes and their behavioral consequences. However, the model should not be interpreted as fully recapitulating either the phenotype of naturally aged tree shrews or the complete pathological course of human AD.

## Data Availability

The data generated and analyzed during this study are available from the corresponding author upon reasonable request. The human APP reference sequence used for vector design is publicly available from NCBI RefSeq (NM_000484.4), and the corresponding protein information is available from UniProt (P05067).

## Supplementary Materials

The following supporting information can be downloaded at: https://www.mdpi.com/article/10.3390/biology15131071/s1. Figure S1: Preliminary verification of the CA1-based stereotaxic injection target in tree shrews by anatomical atlas comparison and dye-injection experiments. Figure S2: Original unprocessed PCR gel image for verification of exogenous hAPP in hippocampal tissues. Figure S3: RT-qPCR amplification and melt peak curves for the reference genes GAPDH and HPRT1. Figure S4: Original uncropped Western blot images for Aβ40, Aβ42, 4G8, total Aβ, and β-actin. Table S1: Primer sequences and amplicon sizes used for PCR verification and RT-qPCR. Table S2: RT-qPCR primer amplification efficiencies and standard-curve R^2^ values. Table S3: Effect-size estimates for behavioral outcomes, including novel object recognition, locomotor measures, social approach, and social novelty behavior. Table S4: Effect-size estimates for primary histopathological outcomes, including Thioflavin S-reactive aggregate-associated signals, AT8 immunoreactivity, GFAP and Iba-1 reactivity, and synaptic marker immunoreactivity.

## Abbreviations

The following abbreviations are used in this manuscript:

AD	Alzheimer’s disease
Aβ	amyloid-β
APP	amyloid precursor protein
hAPP	human amyloid precursor protein
tsAPP	tree shrew amyloid precursor protein
AAV	adeno-associated virus
hAPP-SLA	human APP carrying Swedish, London, and Austrian mutations
