# Supplementary material for: Establishment and Characterization of an Aβ-Related Alzheimer’s Disease-like Tree Shrew Model Following CA1-Coordinate–Directed Stereotaxic AAV Delivery of Human Triple-Mutant APP
Source: Biology (Basel). 2026 Jul 4;15(13):1071. doi: 10.3390/biology15131071 (PMC13359454; doi:10.3390/biology15131071)
Supplement: Supplementary file 1 [file biology-15-01071-s001.zip › biology-4372593-supplementary.pdf]

Supplementary Materials

Supplementary Information for
Establishment and Characterization of an Aβ-Related Alzheimer's Disease-like Tree Shrew Model by AAV-Mediated Hippocampal Delivery of Human Triple-Mutant APP

Supplementary Figure S1. Preliminary verification of the CA1-based stereotaxic injection target in tree shrews. (a–c) Coronal atlas section and representative preliminary dye-injection images obtained at AP −4.15 mm, ML ±5.10 mm, and DV −4.10 mm. (d–f) Coronal atlas section and representative preliminary dye-injection images obtained at AP −4.82 mm, ML ±6.10 mm, and DV −6.50 mm. (g–i) Coronal atlas section and representative preliminary dye-injection images obtained at AP −5.36 mm, ML ±6.50 mm, and DV −11.60 mm. The final coordinates used for bilateral AAV injection were AP −5.36 mm, ML ±6.50 mm, and DV −11.60 mm.

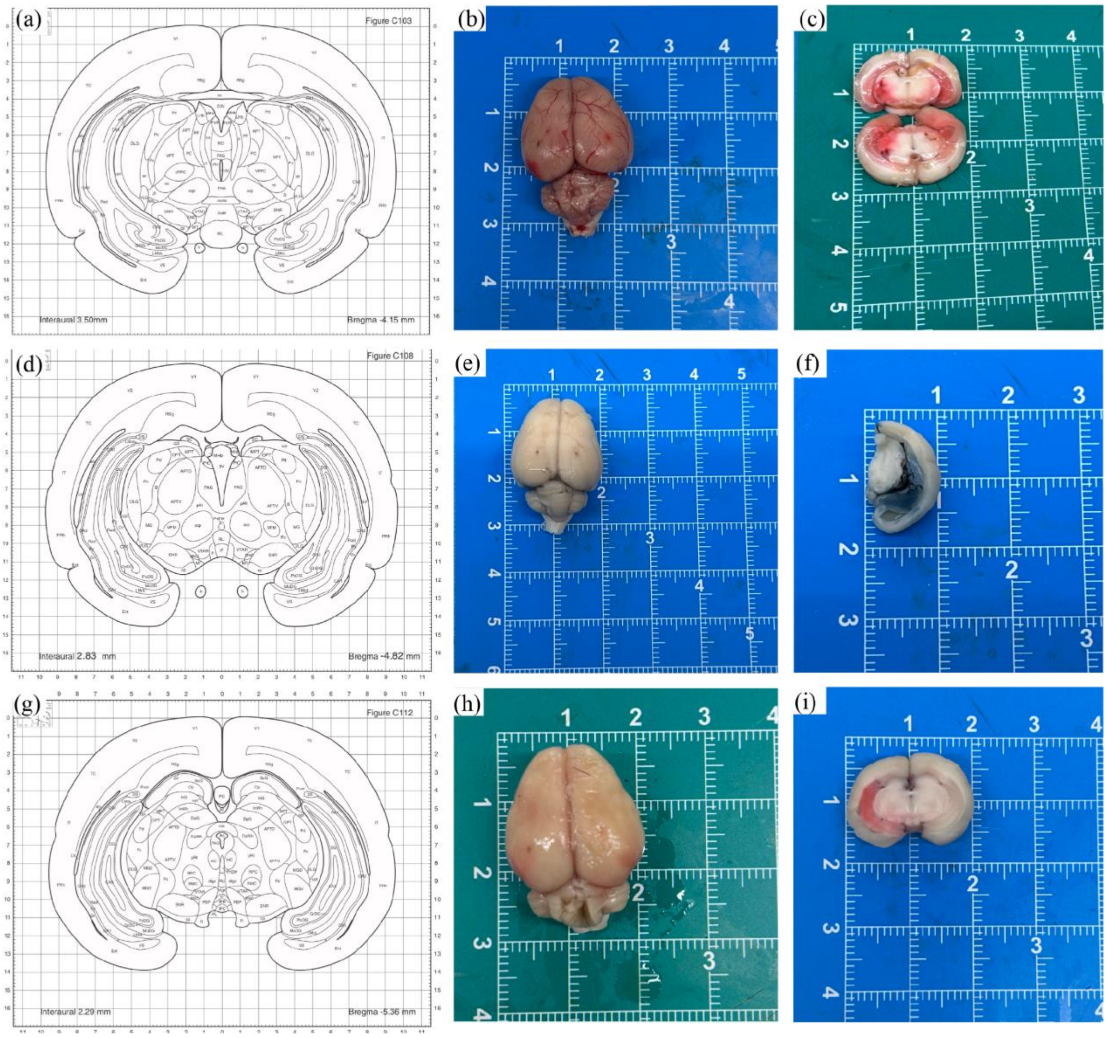

Supplementary Figure S2. Original unprocessed PCR gel image for verification of exogenous hAPP in hippocampal tissues. M, DNA marker. The expected hAPP amplicon size was 726 bp. Lane identities are indicated on the gel image.

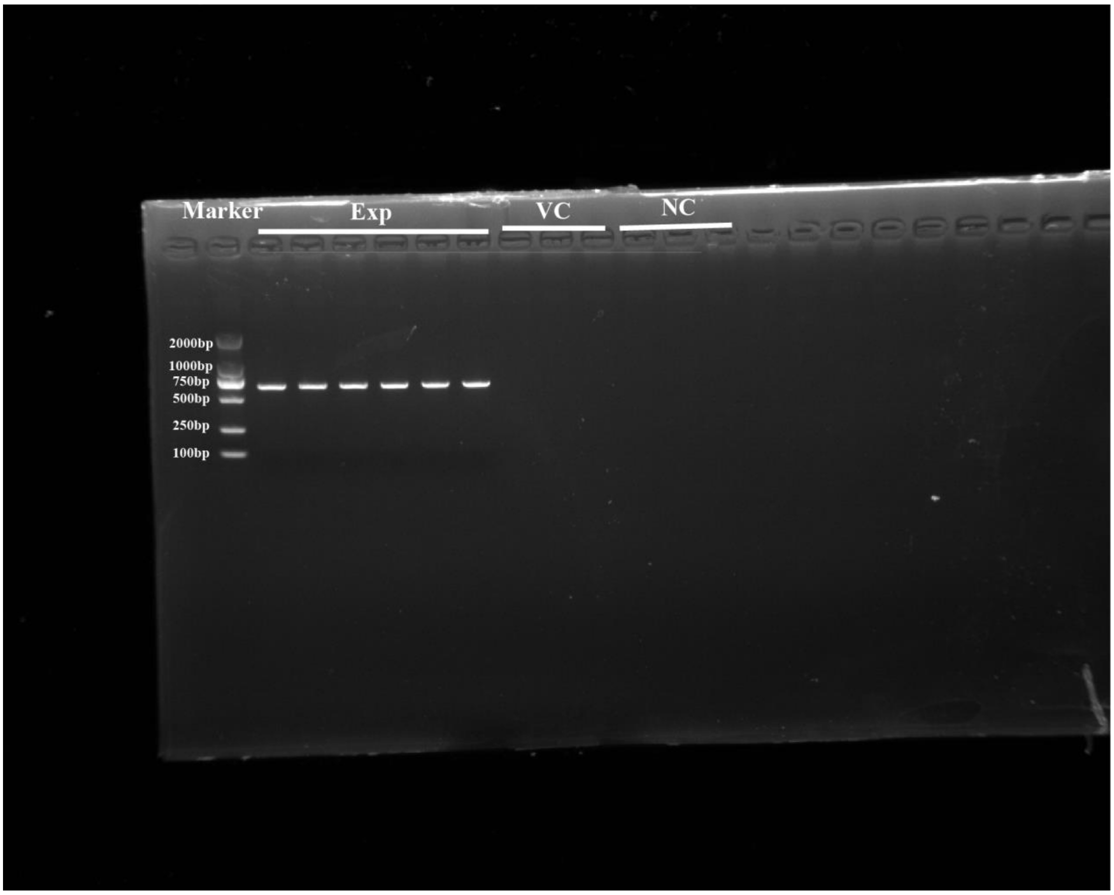

Supplementary Figure S3. RT-qPCR amplification and melt peak curves for the reference genes GAPDH and HPRT1. (A) Amplification curves for GAPDH. (B) Melt peak curves for GAPDH. (C) Amplification curves for HPRT1. (D) Melt peak curves for HPRT1. GAPDH and HPRT1 were used jointly as reference genes for RT-qPCR normalization.

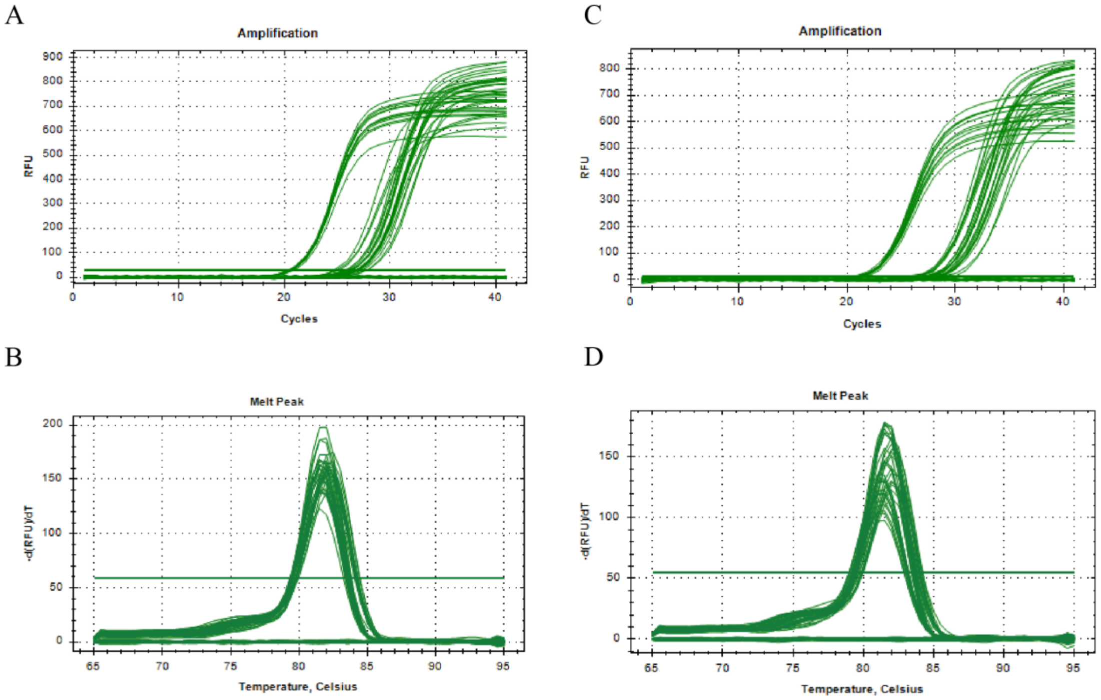

Supplementary Figure S4. Original uncropped Western blot images corresponding to Figure 4E. (A) $A\beta_{40}$ and the corresponding β-actin blot. (B) $A\beta_{42}$ and the corresponding β-actin blot. (C) 4G8-reactive APP/Aβ-related signal and the corresponding β-actin blot. (D) Total Aβ and the corresponding β-actin blot. Red boxes indicate the bands used for densitometric quantification in the main text. The lane order was consistent with that shown in Figure 4E, and sample identities are indicated above or below the corresponding lanes.

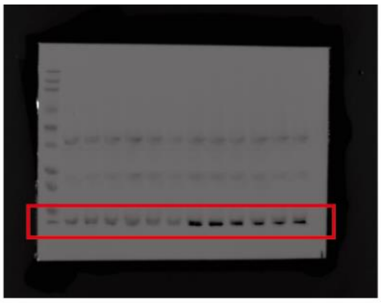

Figure 4E Aβ40

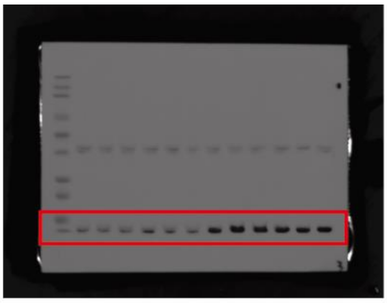

Figure 4E Aβ42

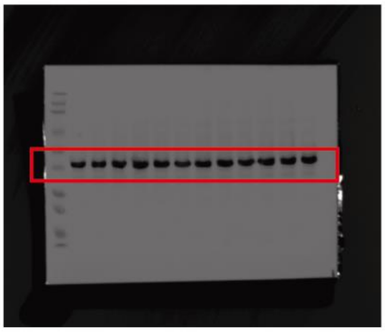

Figure 4E Aβ40 β-actin

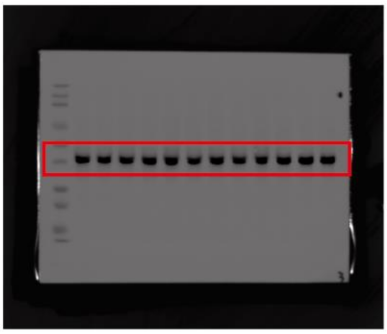

Figure 4E Aβ42 β-actin

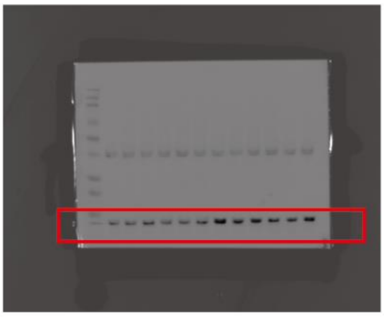

Figure 4E 4G8

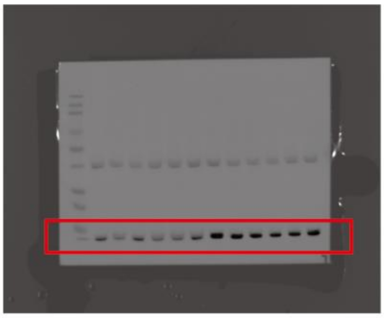

Figure 4E TotalAβ

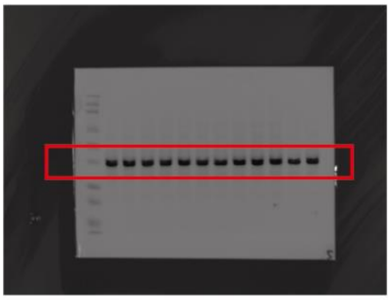

Figure 4E 4G8 β-actin

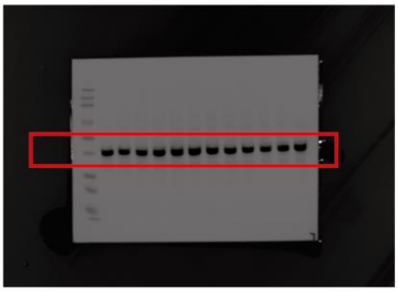

Figure 4E TotalAβ β-actin

Supplementary Table S1. Primer sequences used for PCR verification and RT-qPCR.

Application	Gene/target	Species/ template	Forward primer (5′–3′)	Reverse primer (5′–3′)	Amplicon size (bp)
PCR verification	hAPP	Human APP/AAV vector	CCGCGCAGAAC AGAAGGACA	GCGTCAACCTC CACCACACC	726
RT-qPCR	hAPP	Human APP	TGGAGGTACCC ACTGATGGT	GCAGGATGCCT TCCTTGGTA	159
RT-qPCR	tsAPP	Tree shrew	ACATGGCCTCC AAGGAGTAGA	GGAGGGGAGC TTCTCAGTGT	124
RT-qPCR	GAPDH	Tree shrew	GAGGTGGTTCG AGTTCCTACA	TTGACGTTCTG CCTCTTCCC	180
RT-qPCR	HPRT1	Tree shrew	CATTTTGGCCG CTACCACTC	ACCCTTTCCAA ATCCTGGGC	155

Note: hAPP-SLA refers to human APP carrying Swedish, London, and Austrian mutations. tsAPP refers to endogenous tree shrew APP. Primer efficiency and R^2 values were calculated from standard curves generated using serially diluted cDNA templates. GAPDH and HPRT1 were used as reference genes for RT-qPCR normalization.

Supplementary Table S2. Validation parameters of RT-qPCR primers.

Gene/target	Efficiency (%)	R^2	Purpose
hAPP	96.00	0.9996	Detection of exogenous human mutant APP expression
tsAPP	94.40	0.9977	Detection of endogenous tree shrew APP expression
GAPDH	99.85	0.9950	Reference gene
HPRT1	101.57	0.9986	Reference gene

Table S3. Effect-Size Estimates for Primary Behavioral Outcomes

Outcome	Statistical analysis	Omnibus result	Effect size	Key pairwise comparisons
NOR index, exploration time	Ordinary one-way ANOVA with Tukey's multiple-comparisons test	$F(3,20) = 16.87$, $p < 0.0001$	$\eta^2 = 0.717$	EXP vs. VC: adjusted $p < 0.0001$, Hedges' $g = -3.278$; EXP vs. AGED: adjusted $p = 0.0003$, Hedges' $g = -2.737$
NOR index, entries	Ordinary one-way ANOVA with Tukey's multiple-comparisons test	$F(3,20) = 40.30$, $p < 0.0001$	$\eta^2 = 0.858$	EXP vs. VC: adjusted $p < 0.0001$, Hedges' $g = -4.823$; EXP vs. AGED: adjusted $p < 0.0001$, Hedges' $g = -5.009$
NOR index, total distance	Ordinary one-way ANOVA with Tukey's multiple-comparisons test	$F(3,20) = 17.13$, $p < 0.0001$	$\eta^2 = 0.720$	EXP vs. VC: adjusted $p = 0.9789$, Hedges' $g = -0.209$; AGED vs. VC: adjusted $p < 0.0001$, Hedges' $g = -3.200$
Social approach interaction time, Empty versus Stranger 1	Two-way repeated-measures ANOVA	Group × stimulus interaction: $F(3,20) = 7.062$, $p = 0.0020$	partial $\eta^2 = 0.514$	Primary effect-size estimate: group × stimulus interaction (partial $\eta^2 = 0.514$).
Social approach preference percentage	Ordinary one-way ANOVA with Dunnett's multiple-comparisons test	$F(3,20) = 8.286$, $p = 0.0009$	$\eta^2 = 0.554$	EXP vs. NC: adjusted $p = 0.0056$, Hedges' $g = -1.887$; AGED vs. NC: adjusted $p = 0.0073$, Hedges' $g = -1.824$
Social novelty interaction time, Stranger 1 versus Stranger 2	Two-way repeated-measures ANOVA	Group × stimulus interaction: $F(3,20) = 7.928$, $p = 0.0011$	partial $\eta^2 = 0.543$	Primary effect-size estimate: group × stimulus interaction (partial $\eta^2 = 0.543$).
Social novelty preference percentage	Ordinary one-way ANOVA with Dunnett's multiple-comparisons test	$F(3,20) = 6.763$, $p = 0.0025$	$\eta^2 = 0.504$	EXP vs. NC: adjusted $p = 0.0102$, Hedges' $g = -1.746$; AGED vs. NC: adjusted $p = 0.0102$, Hedges' $g = -1.746$
Total interaction distance in the three-chamber test	Ordinary one-way ANOVA with Tukey's multiple-comparisons test	$F(3,20) = 1.823$, $p = 0.1754$	$\eta^2 = 0.215$	EXP vs. VC: adjusted $p = 0.9507$, Hedges' $g = -0.282$; AGED vs. VC: adjusted $p = 0.1497$, Hedges' $g = -1.187$

Notes: All groups included six animals (n = 6 per group). NOR, novel object recognition; NC, normal control; VC, vector control; EXP, AAV-hAPP-SLA-treated group; AGED, naturally aged group. For ordinary one-way ANOVA, η^2 was calculated as $SS_{between}/SS_{total}$ and corresponds to the R squared value reported by Prism. For two-way repeated-measures ANOVA, partial η^2 is reported for the group × stimulus interaction, which represents differences among groups in stimulus preference patterns. Adjusted p-values were obtained using Tukey's, Dunnett's, or Sidak's multiple-comparisons tests, as indicated. Hedges' g values are signed according to the order of the listed comparison (group 1 minus group 2); therefore, a negative value indicates that the first-listed group had a lower value than the second-listed group. For interaction-time outcomes, the primary effect-size estimate was the group × stimulus interaction rather than a single independent-group pairwise effect size.

Table S4. Effect-Size Estimates for Primary Histopathological Outcomes

**Outcome**	**Statistical analysis**	**Omnibus result**	**Effect size**	**Key pairwise comparisons**
Thioflavin S-positive area (%)	with Games–Howell multiple-comparisons test	$F(3,10.12) = 107.86$, $p < 0.0001$	$\eta^2 = 0.799$	EXP vs. VC: adjusted $p < 0.001$, Hedges' $g = 8.408$; AGED vs. VC: adjusted $p = 0.0169$, Hedges' $g = 2.425$
AT8-positive area (%)	Welch's ANOVA with Games–Howell multiple-comparisons test	$F(3,10.19) = 47.53$, $p < 0.0001$	$\eta^2 = 0.849$	EXP vs. VC: adjusted $p < 0.001$, Hedges' $g = 5.772$; AGED vs. VC: adjusted $p = 0.0039$, Hedges' $g = 3.550$
GFAP-positive area (%)	Ordinary one-way ANOVA with Tukey's multiple-comparisons test	$F(3,20) = 162.5$, $p < 0.0001$	$\eta^2 = 0.961$	EXP vs. VC: adjusted $p < 0.0001$, Hedges' $g = 10.248$; AGED vs. VC: adjusted $p < 0.0001$, Hedges' $g = 3.300$
Iba-1-positive area (%)	Ordinary one-way ANOVA with Tukey's multiple-comparisons test	$F(3,20) = 40.46$, $p < 0.0001$	$\eta^2 = 0.859$	EXP vs. VC: adjusted $p < 0.0001$, Hedges' $g = 3.627$; AGED vs. VC: adjusted $p < 0.0001$, Hedges' $g = 3.159$
Synaptophysin-positive area (%)	Ordinary one-way ANOVA with Tukey's multiple-comparisons test	$F(3,20) = 10.03$, $p = 0.0003$	$\eta^2 = 0.601$	EXP vs. VC: adjusted $p = 0.0040$, Hedges' $g = -2.105$; AGED vs. VC: adjusted $p = 0.0002$, Hedges' $g = -2.779$; AGED vs. NC: adjusted $p = 0.0497$, Hedges' $g = -1.453$
PSD-95-positive area (%)	Ordinary one-way ANOVA with Tukey's multiple-comparisons test	$F(3,20) = 20.62$, $p < 0.0001$	$\eta^2 = 0.756$	EXP vs. VC: adjusted $p = 0.0052$, Hedges' $g = -2.048$; AGED vs. VC: adjusted $p < 0.0001$, Hedges' $g = -3.310$

Notes: All groups included six animals (n = 6 per group). NC, normal control; VC, vector control; EXP, AAV-hAP P-SLA-treated group; AGED, naturally aged group. For ordinary one-way ANOVA, η^2 was calculated as $SS_{between}/SS_{total}$ and corresponds to the R squared value reported by Prism. For outcomes analyzed using Welch's ANOVA, η^2 was retained as a d escriptive measure of the proportion of total variance attributable to group differences, calculated from the conventional betw een-group and total sums of squares. Games–Howell multiple-comparisons tests were applied when homogeneity of variance was not satisfied; otherwise, Tukey's multiple-comparisons test was used. Hedges' g values are signed according to the orde r of the listed comparison, with negative values indicating lower values in the first-listed group than in the second-listed grou p. Adjusted p-values refer to the post hoc multiple-comparisons tests indicated in the table.
